# Public and Private Nursing Institutions

**Published:** 1887-05-28

**Authors:** 


### PUBLIC AND PRIVATE NURSING INSTITUTIONS.

By a Member of the London Association of Nurses.

Speaking for myself only, I heartily thank Miss Manson for saying that the terms of the London Association of Nurses are liberal. I have often heard private patients and their friends regret the small amount a nurse receives in comparison to the fees which are paid for her. It may be of interest to some just now to hear that when sending to 62, New Bond Street for a nurse, she will in all probability be the chief one to benefit by their doing so. As the matron of a large hospital, I suppose Miss Manson will allow that, even after a careful training, all nurses do not possess the same qualifications; but, to make us a little more alike than we are now, I would urge that a Register of Nurses be kept at Norfolk House, Norfolk Street, of all trained nurses—those who may be still nursing in hospitals, as well as those who are working for institutions, or nursing cases for themselves. Only it must not be forgotten by those who draw up the rules, that the training schools and hospitals do not generally give certificates to their nurses, only to those probationers or pupils who pay for a year's training. I, and a hundred more no doubt, could only show by a reference that we had been at such a place for such a time. Is not Miss Manson misleading people when she says, "St. Bartholomew's, St. Thomas's, and Guy's have fixed the complete term of training at a period of not less than three years"? She cannot mean that a nurse is under the eye of a superintendent or sister for the whole of this term. Do they not go private nursing sometimes? or at St. Thomas's perhaps to help at some infirmary?

If the Matron of St. Bartholomew's really thinks the time is near when all private nursing will be done by the hospitals—for which, of course, they will take the lion's share of the fees—then I hope she will forward a National Pension Fund for Nurses with as much zeal as she has raised another question,—for we have not all the happy prospect of wedded bliss close at hand.

This is much needed, and I for one should be glad to see it started.

I enclose my name and address, but, for obvious reasons, not for publication.

